# Impact of Cadmium on Prostate-Specific Antigen and Endothelial Markers: A Risk for Prostatic Damage

**DOI:** 10.3390/toxics13121049

**Published:** 2025-12-04

**Authors:** Servet Birgin İritaş, Melih Gaffar Gözükara, Lütfiye Tutkun, Deniz Özkan Vardar, Murat Büyükşekerci, Gülsüm Abusoğlu, Serdar Deniz, Vugar Ali Türksoy, Engin Tutkun

**Affiliations:** 1 Department of Forensic Medicine, Faculty of Medicine, Ankara Yildirim Beyazit University, 06800 Ankara, Türkiye; 2 Department of Public Health, Faculty of Medicine, Ankara Yildirim Beyazit University, 06800 Ankara, Türkiye; mggozukara@aybu.edu.tr; 3 The Association of Industrial Toxicology and Occupational Hygiene, 06680 Ankara, Türkiye; lutfiye.tutkun@etok-ihider.org (L.T.); engin.tutkun@etok-ihider.org (E.T.); 4 Vocational School of Health Services, Pharmacy Services, Lokman Hekim University, 06510 Ankara, Türkiye; deniz.ozkanvardar@lokmanhekim.edu.tr; 5 Department of Pharmacology, Ankara Ataturk Sanatorium Training and Research Hospital, 06290 Ankara, Türkiye; murat.buyuksekerci@saglik.gov.tr; 6 Vocational School of Health Sciences, Selcuk University, 42130 Konya, Turkey; gulsum.tekin@selcuk.edu.tr; 7 Department of Public Health, Malatya Turgut Özal University, 44900 Malatya, Türkiye; serdar.deniz@ozal.edu.tr; 8 Department of Public Health, Yozgat Bozok University, 66900 Yozgat, Türkiye; v.aliturksoy@bozok.edu.tr

**Keywords:** cadmium, ADMA, SDMA, nitric oxide, PSA, endothelial dysfunction, occupational exposure

## Abstract

Cadmium (Cd) is a persistent toxic metal that bioaccumulates in human tissues and may disrupt redox and endocrine pathways, yet the metabolic mechanisms linking Cd exposure to both endothelial and prostate dysfunctions remain insufficiently defined. This study investigated whether chronic occupational Cd exposure alters methylated arginine metabolism and prostate-specific antigen (PSA) levels, indicating a shared toxicometabolic axis. A total of 150 male workers were enrolled, including 75 metallurgical employees with documented Cd exposure and 75 matched controls. All participants were non-smokers, eliminating confounding from tobacco-related oxidative or endocrine effects. Urinary Cd concentrations were quantified using Inductively Coupled Plasma–Mass Spectrometry (ICP–MS), and serum asymmetric dimethylarginine (ADMA), symmetric dimethylarginine (SDMA), L-arginine, citrulline, and PSA were measured by Liquid Chromatography Tandem Mass Spectrometry (LC-MS/MS) and electrochemiluminescence. The use of Inductively Coupled Plasma–Mass Spectrometry for cadmium quantification and LC-MS/MS for methylated arginine profiling provided high analytical specificity and sensitivity, strengthening the validity of biomarker measurements. Correlation and multivariable analyses adjusted for age and body mass index. Cd-exposed workers demonstrated significantly elevated urinary Cd, PSA, ADMA, and SDMA levels, alongside reduced arginine/ADMA ratios, consistent with impaired nitric oxide bioavailability. Urinary Cd strongly correlated with PSA and ADMA levels. These findings indicate that Cd may disrupt the nitric oxide pathway and elevates PSA, supporting a mechanistic link between vascular and prostate stress. Combined ADMA, SDMA, and PSA profiling may serve as an early biomarker panel for Cd-related metabolic injury in occupational settings.

## 1. Introduction

Prostate cancer (PCa) and diseases represent a significant public health challenge, representing the second most common cancer among men in the world [1]. The incidence of PCa and diseases varies geographically, with lower incidence in Asian populations and greater incidence in the West, but environmental and metabolic modifying influences are increasingly recognized as significant contributors to the development of the disease [2,3]. Known risk determinants include obesity, androgenic imbalance, and dietary factors such as selenium, lycopene, vitamin D/E, and fat intake, though nutrition appears to have only a limited effect on PSA levels [4,5]. More recently, the focus has evolved to include the potential role of heavy metals, particularly cadmium (Cd), in the etiology of prostate diseases.

Cd is a naturally occurring, biologically non-essential heavy metal with a long biological half-life (10–30 years), allowing it to bioaccumulate in the soft tissues of the liver, kidney, and prostate. Major sources of exposure include the contaminated food chain, industrial waste, and cigarette smoke, but occupationally, major exposure occurs in the battery manufacturing, pigment, electroplating, and metal smelting industries [6]. The inhalation of Cd-laden dust or fumes allows for rapid pulmonary absorption and systemic distribution, resulting in oxidative stress, impaired deoxyribonucleic acid (DNA) repair, and disruption of zinc (Zn) dependent enzymes and transcription factors [7].

Epidemiological data relevant to Cd exposure and risk of prostate disease has been inconsistent. Some reports of exposure to Cd in industrial occupations have reported an elevated incidence of prostate disease morbidity, while other reports show no correlation [8,9]. However, there is accumulating evidence to support the concept that Cd may be a prostate-selective endocrine disruptor, affecting androgen receptor signaling, and may reflect early prostatic stress or dysregulation [10,11,12].

In addition to its potential for carcinogenicity, Cd exposure has been associated with impairment of endothelium function and cardiovascular disease (CVD) [13]. Mechanistically, Cd fosters the generation of reactive oxygen species, metallothionein sequestration of Zn, and the inhibition of the nitric oxide (NO) pathway [14,15]. Similar oxidative-stress–mediated endocrine disturbances have been observed with other environmental toxicants, which disrupt hormonal homeostasis through redox-related damage to regulatory axes, and could point to the biological plausibility of Cd-related vascular and endocrine alterations [16]. Increased serum levels of methylated arginine derivatives, asymmetric dimethylarginine (ADMA), and symmetric dimethylarginine (SDMA) can potentiate the inhibition of NO production and represent validated predictors of endothelial dysfunction and cardiovascular risk [17].

Given the interrelated mechanisms involving oxidative stress, endothelial dysfunction, and prostate pathophysiology, this study will assess the effects of Cd exposure through occupation on serum levels of ADMA and prostate-specific antigen (PSA), which represent vascular and prostatic-specific responses, respectively. In the present work, metabolic and endocrine biomarkers will be correlated in order to evaluate the two potentially toxicometabolic pathways that Cd might induce to result in both endothelial impairment and prostatic dysfunction. Despite accumulating evidence on Cd-mediated endocrine and vascular toxicity, no prior study has simultaneously examined both endothelial methylarginine dysregulation (ADMA, SDMA) and prostate-specific activation (PSA) within the same occupationally exposed human cohort [18,19]. This represents a critical research gap, as endothelial dysfunction and prostatic stress may share upstream Cd-induced metabolic pathways. Therefore, our study integrates these two biomarker domains to delineate a potential shared toxicometabolic axis.

Despite indications of exposure-oxidative stress connections associated with prostatic and endothelial pathology, to our knowledge, there have been no prior human studies that simultaneously investigate endothelial methylarginine dysregulation (ADMA, SDMA) and prostate-activation (PSA) within a single occupationally exposed cohort in humans, with most prior research examining these pathways in isolation. Integrating some aspects of the understanding of Cd toxicity with others from our studies raises a question: are vascular injury signals and prostate stress markers co-seamless in their connection, are they entirely different, or are there separate consequences of Cd toxicity? Oxidative stress, nitric oxide pathway disruption, Zn Zn-dependent hormonal interference may affect widespread endothelial as well as prostatic alteration through a common upstream pathway. In this light, we highlight links we made in the exposure of an entire cohort that faces exposure.

## 2. Materials and Methods

### 2.1. Study Groups

This cross-sectional analytical study utilized a total of 150 male subjects, including 75 Cd-exposed workers and 75 non-exposed controls. Cd-exposed workers were all non-smokers working at a single metallurgical plant that has historically been known for Cd-exposure to its workers. Controls were administrative/technical personnel employed by the same organization with no occupational exposure to Cd or other heavy metals. Inclusion of the two populations within the same industrial environment minimized confounding variables due to socio-economic status and/or environmental exposures. Written informed consent was collected from all subjects before enrollment into this study.

In addition to reducing variability and ensuring homogeneity between subjects, stringent selection criteria were used to minimize bias. Subjects were included if they were male, aged 25–60 years, had at least one year of service in their current job, were non-smokers and non-drinkers, and demonstrated the absence of chronic cardiovascular, renal, hepatic, metabolic, respiratory, or endocrine diseases (including ischemic heart disease, hypertension, diabetes mellitus, thyroid disorders, and chronic kidney disease), absence of active infections, absence of malignancy, absence of prostate pathology, absence of recent exposure to other heavy metals, organic solvents or environmental pollutants outside the workplace, absence of multivitamin supplements such as Zn, selenium etc., and absence of use of medications that influence vascular or hormonal regulation (e.g., statins, antihypertensives, corticosteroids, or hormone-replacement therapies). All participants worked in the same industrial sector with similar cadmium exposure patterns and fixed-shift schedules, which limited potential lifestyle-related variability across the cohort.

Each participating worker completed an anonymous questionnaire detailing their work duration, work department, protective equipment usage, and any possible Cd exposure from non-work-related activities. Urinary Cd concentration (measured via ICP-MS) served as the major bioindicator of Cd accumulation in the body, and was measured in two ways: (a) µg/L and (b) µg/g creatinine (after adjusting for creatinine levels). Workers with urinary Cd levels > than the Biological Exposure Index value of 5 µg/g creatinine [20] were classified as having high exposure levels.

### 2.2. Sampling and Biochemical and Analytical Methods

#### 2.2.1. Sample Collection and Preparation

Between 08:00 and 10:00 a.m., after an overnight fast to decrease circadian rhythm and dietary effects on metabolic parameters, although it has a minuscule effect, venous blood and spot urine samples were collected from all participants.

Trace-element-free vacutainers were used to collect samples to minimize contamination of the trace element levels. To further reduce the risk of trace element contamination, serum samples were collected in polypropylene tubes. Samples were then centrifuged for 10 min at 3000 revolutions per minute. to separate the serum portion from red cells. Aliquots of the serum samples were prepared for subsequent measurement of hormones and metabolites.

Spot urine samples were immediately acidified with ultrapure nitric acid (1% *v*/*v*) and then stored at −80 °C. All reagents and consumables used were either of analytical or High-Performance Liquid Chromatography grade.

#### 2.2.2. Measurement of Urinary Cd

The concentration of Cd in urine was measured by Inductively Coupled Plasma–Mass Spectrometry (ICP-MS) (Agilent 7700×, Agilent Technologies, Tokyo, Japan). Standards of the multi-element calibration solution, National Institute of Standards and Technology Standard Reference Material 1643f, were used for calibration purposes, while the internal standardization method with rhodium (Rh) was employed to correct for instrument drift. The limit of detection for Cd was 0.05 µg/L, and the limit of quantitation for Cd was 0.15 µg/L. Using urine reference materials of known quality control, such as Seronorm™ Trace Elements (Sero AS, Hvalstad, Norway), analytical accuracy was confirmed to be greater than 95%, but less than 105% recovery rate. Urinary Cd concentrations were initially quantified in µg/L and subsequently normalized to urinary creatinine to obtain creatinine-adjusted values expressed as µg/g creatinine.

#### 2.2.3. Quantification of Amino Acids and Methylated Arginine Derivatives

Using Liquid Chromatography–Tandem Mass Spectrometry (LC-MS/MS) (AB Sciex 4500 QTrap, Foster City, CA, USA), plasma concentrations of L-arginine, homoarginine, citrulline, ADMA, and SDMA were analyzed. Methanol with isotopically labeled internal standards ([L-arginine-^13C6], [ADMA-d6], [SDMA-d7]) was added to the sample to precipitate proteins. Following centrifugation and injection into the LC-MS/MS apparatus, the sample underwent chromatographic separation on a Phenomenex Luna C18 column (2.1 × 100 mm, 3 µm) employing a mobile phase that consisted of 0.1% formic acid in water and acetonitrile under gradient elution conditions at a flow rate of 0.25 mL/min. Positive electrospray ionization mode was utilized to analyze the sample in multiple-reaction-monitoring mode to quantify amino acids. Calibration curves were linear over the physiological range (r^2^ > 0.995), and intra- and inter-assay coefficients of variation for all analytes were less than 8%.

L-arginine, homoarginine, citrulline, ADMA, and SDMA were selected because they represent key nodes of the nitric oxide (NO) pathway. ADMA and SDMA are well-validated endogenous inhibitors of NO production and established biomarkers of endothelial dysfunction. Arginine serves as the substrate for NO synthesis, while citrulline reflects downstream NO cycle turnover. Homoarginine was included due to its role as an alternative substrate for nitric oxide synthase and its known inverse relationship with cardiovascular risk. Together, these metabolites provide a comprehensive biochemical profile of Cd-related alterations in the arginine–NO axis.

#### 2.2.4. PSA Measurement

PSA was tested by electrochemiluminescence immunoassay on a Cobas e601 Analyzer (Roche Diagnostics, Mannheim, Germany). All tests were performed in duplicate, in accordance with the instructions from the manufacturer. Internal quality controls were applied every day. The laboratory takes part in external quality assurance (External Quality Assurance Scheme, Randox International Quality Assessment Scheme) programs for trace elements and hormones.

#### 2.2.5. Quality Assurance and Data Validation

Analytical quality was verified by the addition of pooled control samples in each run. The calibration of the instrument and the use of a blank control were repeated after every 10 samples in order to check possible carryover or drift. Outliers exceeding ±3 standard deviation (SD) from the group mean were reanalyzed in order to confirm validity. All measured concentrations are presented as SI units, and the trace element determination data were corrected for urinary creatinine in order to allow comparison.

### 2.3. Statistical Analysis

Statistical analyses were performed using the IBM Statistical Package for Social Sciences Statistics version 20.0 (Armonk, NY, USA, IBM Corporation). All continuous variables were analyzed for their distributions by means of the Shapiro–Wilk test for normality and the Levene’s test to analyze the homogeneity of variances. Since each primary variable met these assumptions, only parametric statistical tests were utilized in this study. All continuous variables were summarized as mean ± SD. Independent-samples *t*-tests were employed for the comparison of exposed subjects and control subjects. Both the Shapiro–Wilk test and Levene’s test were satisfactory for the two groups to be compared. The Pearson Correlation Test was employed to investigate the association of urinary Cd concentrations and biochemical markers (PSA, ADMA, SDMA, Arginine, Citrulline) since they followed the parametric distribution. These are the only parametric methods allowed under the assumption of data distribution. Parametric methods have the advantage of increasing statistical power while decreasing the probability of making Type I or Type II errors.

Using the G*Power 3.1.9.7 software package (Düsseldorf, Germany, Heinrich Heine University), a post hoc power analysis was performed to determine if there was adequate sensitivity for detecting medium-to-large effect sizes for both group comparisons and correlation analyses. The power calculations based on the achieved sample size demonstrated that there was sufficient power for detecting medium-to-large effects for both group comparisons and correlation analyses. Although it was impossible to perform a priori sample size calculation because all eligible and consenting employees within a specific occupation were sampled, the post hoc evaluation confirmed that the sample size was suitable for meeting the study’s objectives.

All statistical analyses were conducted at a two-tailed alpha level of 0.05. GraphPad Prism version 9.0 (San Diego, CA, USA, GraphPad Software) was used to create graphical displays illustrating the differences among groups and the relationships among biomarkers.

## 3. Results

### 3.1. Participant Characteristics

The study examined a total of 150 male subjects, including 75 Cd-exposed workers and 75 non-exposed controls. The demographic and biochemical parameters of the study population are summarized in Table 1. There were no statistically significant differences (*p* = 0.111) in mean age between the two groups, nor were there statistically significant differences (*p* = 0.284) in body mass index (BMI) between the groups, demonstrating good group matching.

### 3.2. Cd Exposure Confirmation

**Figure 1:**
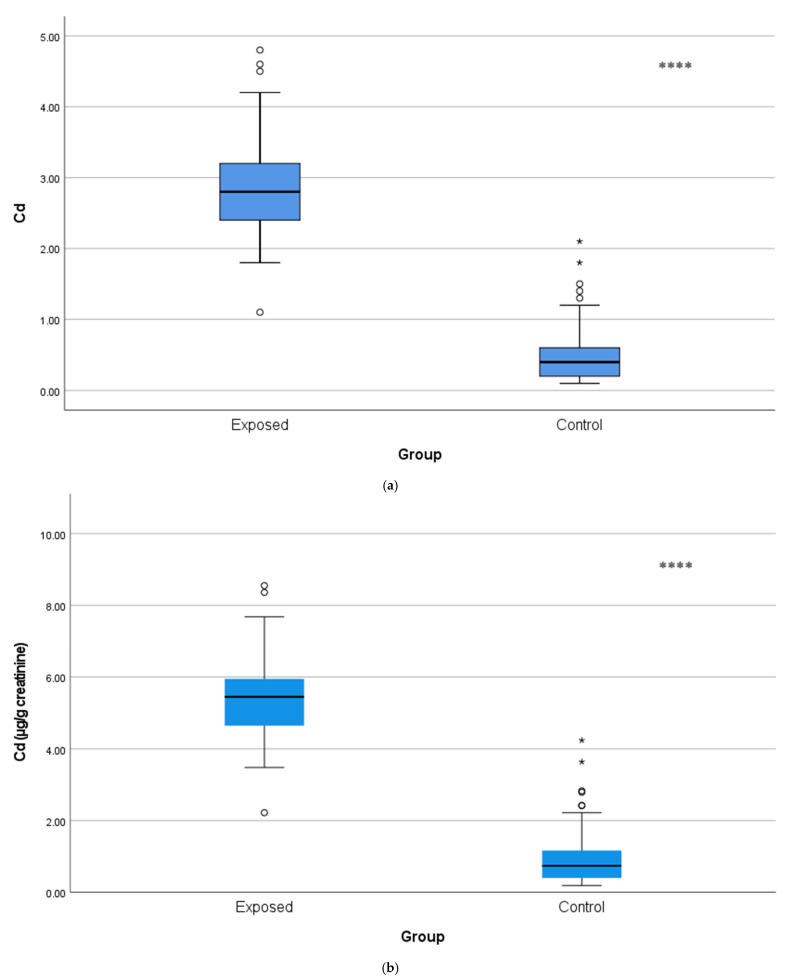
(**a**) Comparison of urine Cd levels with µg/L between Cd-exposed and control groups. The horizontal line inside each box represents the median. The lower and upper edges of the box indicate the 25th and 75th percentiles (IQR). Whiskers extend to 1.5 × IQR. White bullet points (◦) represent outliers (values outside the inner fences, >1.5 × IQR), while a single asterisk above boxes (*) denotes extreme outliers (values beyond the outer fences, >3 × IQR), **** *p* < 0.001. The independent *t*-test was used. (**b**) Comparison of urine Cd levels with µg/g creatinine between Cd-exposed and control groups. Box-plot elements are defined in the legend of (**a**). **** *p* < 0.001. The independent *t*-test was used.

Urinary Cd concentrations were approximately five-fold greater in the exposed workers (2.90 ± 0.72 µg/L or 5.48 ± 1.22 µg/g creatinine) than the control subjects (0.50 ± 0.40 µg/L or 0.97 ± 0.79 µg/g creatinine; *p* < 0.001), as illustrated in Figure 1a,b, thus providing evidence of chronic occupational exposure to Cd.

### 3.3. Prostate Biomarkers

**Figure 2:**
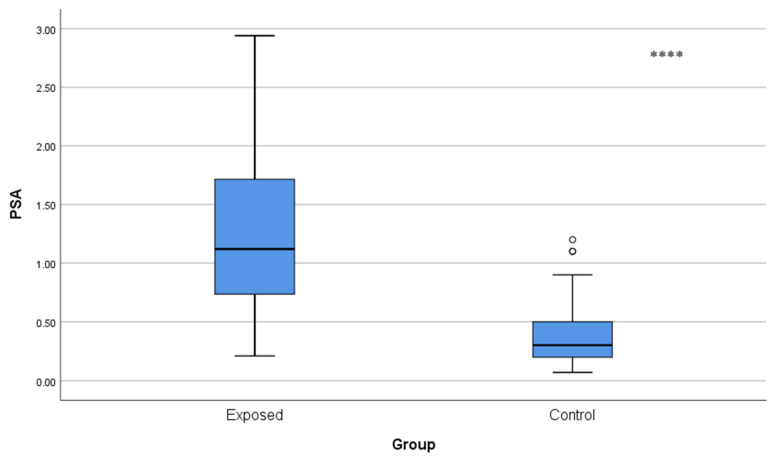
Comparison of blood PSA levels between Cd-exposed and control groups. Box-plot elements are defined in the legend of Figure 1a. **** *p* < 0.001. The independent *t*-test was used.

Serum prostate-specific antigen levels were also significantly greater (*p* < 0.001) in the Cd-exposed cohort (1.27 ± 0.67 ng/mL) than in the control group (0.37 ± 0.25 ng/mL), as depicted in Figure 2.

### 3.4. Arginine and Citrulline Pathway Alterations

**Figure 3:**
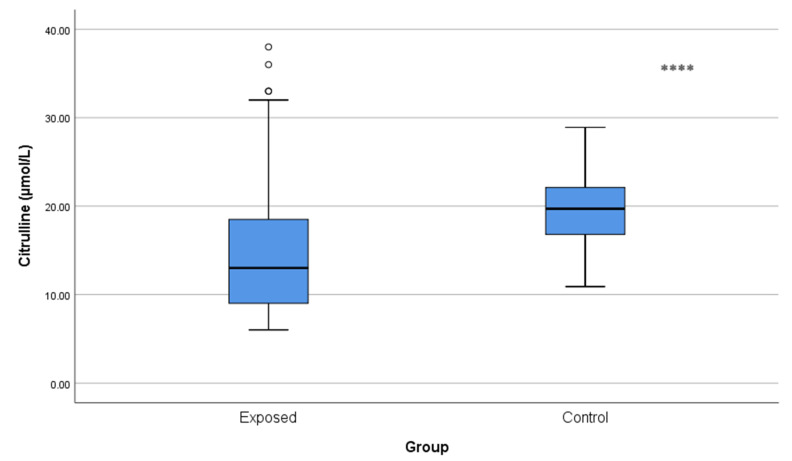
Comparison of citrulline levels between Cd-exposed and control groups. Box-plot elements are defined in the legend of Figure 1a. **** *p* < 0.001. The independent *t*-test was used.

Citrulline concentrations were significantly lower in Cd-exposed workers (15.37 ± 7.99 µmol/L) than in controls (19.59 ± 4.52 µmol/L; *p* < 0.001) (Figure 3), which may indicate an imbalance in urea cycle homeostasis.

### 3.5. Methylated Arginine Biomarkers (ADMA/SDMA)

**Figure 4:**
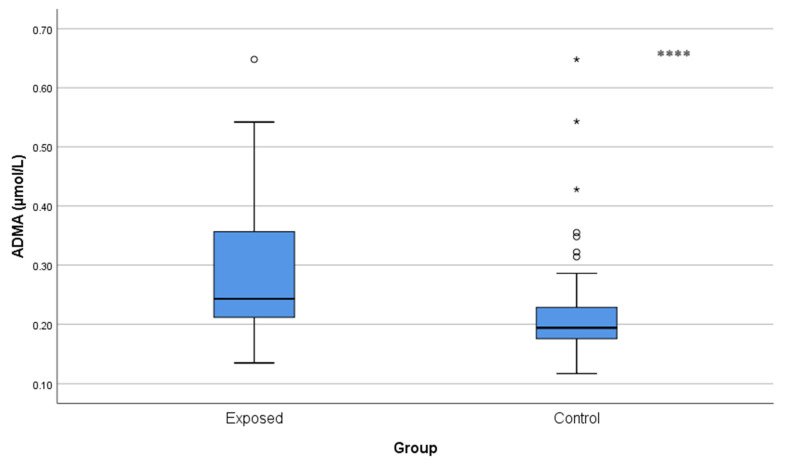
Comparison of ADMA levels between Cd-exposed and control groups. A single asterisk above boxes (*) denotes extreme outliers (values beyond the outer fences, >3 × IQR). Box-plot elements are defined in the legend of Figure 1a. **** *p* < 0.001. The independent *t*-test was used.

**Figure 5:**
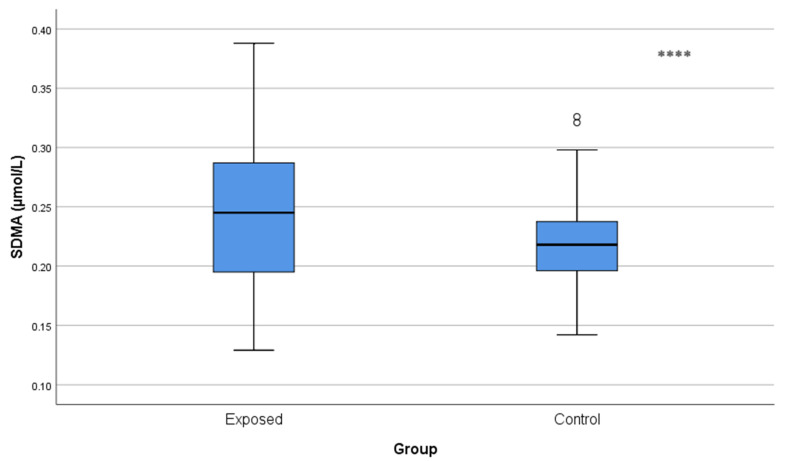
Serum SDMA levels in Cd-exposed versus control groups. Box-plot elements are defined in the legend of Figure 1a. **** *p* < 0.001. The independent *t*-test was used.

ADMA and SDMA concentrations were significantly increased in Cd-exposed workers compared with controls (ADMA: 0.29 ± 0.12 µmol/L vs. 0.22 ± 0.08 µmol/L; *p* < 0.001 and SDMA: 0.25 ± 0.06 µmol/L vs. 0.22 ± 0.04 µmol/L; *p* = 0.001), and indicated a reduction in NO availability (Figure 4 and Figure 5).

### 3.6. NO Bioavailability Indices

**Figure 6:**
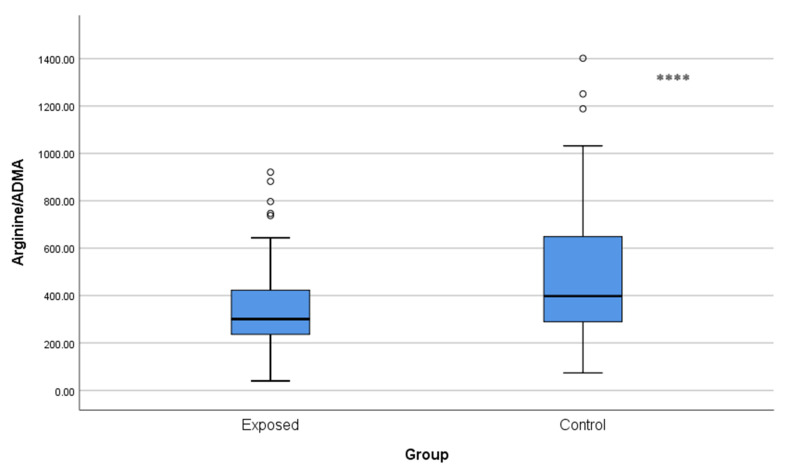
Comparison of arginine/ADMA ratios between Cd-exposed and control groups. Box-plot elements are defined in the legend of Figure 1a. **** *p* < 0.001. The independent *t*-test was used.

The arginine/ADMA ratio was significantly lower in Cd-exposed workers (339.40 ± 182.05) than in controls (483.27 ± 279.19, *p* < 0.001), which may indicate decreased ability of NO to be synthesized (Figure 6).

The SDMA/ADMA ratio was also significantly lower in the Cd-exposed group (0.94 ± 0.28) than in controls (1.09 ± 0.28, *p* = 0.001), which may suggest disrupted pathways of post-translational methylation of arginine.

Plasma arginine concentrations, however, were not found to be significantly different between the Cd-exposed and control groups (87.12 ± 41.91 µmol/L vs. 97.57 ± 59.31 µmol/L, *p* = 0.215) and, thus, could point to Cd-altered post-translational methylation of arginine, but did not alter the levels of the precursor amino acids.

### 3.7. Correlation Analysis

**Figure 7:**
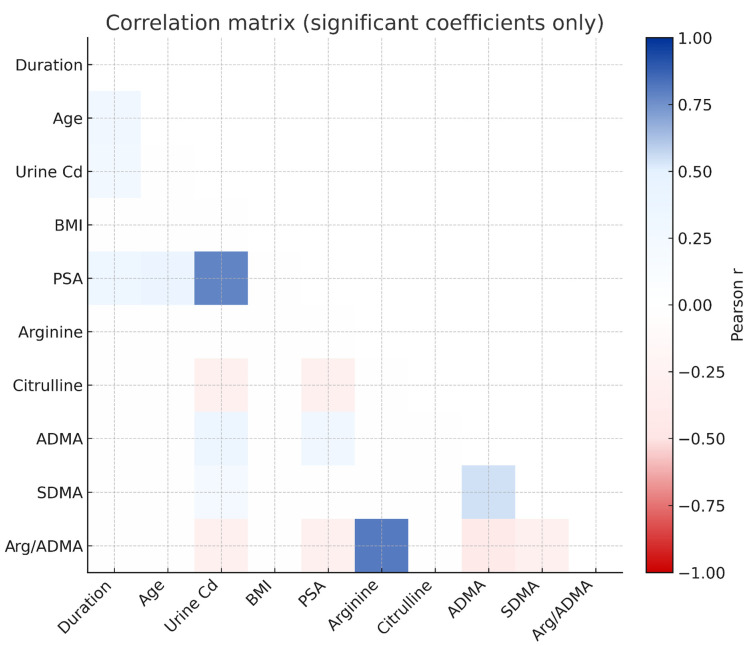
Relationship among occupational Cd exposure, NO pathway metabolites, and prostate biomarkers.

A significant positive association was identified between urinary Cd and PSA (r = 0.783, *p* < 0.001) and also a significant association with ADMA (r = 0.338, *p* = 0.002) and SDMA (r = 0.212, *p* = 0.040). A negative relationship was also identified between urinary Cd and the arginine/ADMA ratio (r = −0.293, *p* = 0.011) may be consistent with a decreased NO-generating capability (Figure 7, Table S1).

Age and duration of Cd exposure were positively correlated with PSA (r = 0.373 and r = 0.300, respectively), but given the weak-to-moderate strength of these associations, the findings should be interpreted as co-variation rather than evidence of an exposure-related accumulation effect. PSA also had a significant positive association with ADMA (r = 0.283, *p* < 0.01) may reflect the inter-relationship of endothelial dysfunction and prostatic dysfunction.

Overall, these results may demonstrate a comprehensive biochemical profile of Cd toxicity, including elevated methylated arginine derivatives, diminished NO bioavailability, and simultaneously altered endocrine and endothelial systems. However, the inferences related to NO remain limited due to the absence of direct measurements.

## 4. Discussion

The current research indicates that chronic occupational Cd exposure may disrupt the metabolic processes of methylated arginine, thereby impairing NO availability and prostate function. Urinary Cd concentration was positively correlated with serum concentrations of ADMA and SDMA, the arginine/ADMA ratio was negatively affected, and PSA was significantly increased in exposed workers. Collectively, these findings demonstrate concurrent alterations in endothelial and prostatic biomarkers in Cd-exposed workers. Additionally, these findings may align with experimental evidence showing that Cd induces oxidative stress–dependent DNA damage that activates the AIM2 inflammasome, leading to IL-1β-driven inflammatory injury [21]. However, these correlations do not establish a shared mechanistic pathway, and because this is a cross-sectional study, causal inference cannot be established. The associations between Cd, PSA, ADMA, and SDMA should be interpreted as correlational rather than mechanistic, and longitudinal studies are required to confirm temporal relationships. The positive correlations among Cd, ADMA, and PSA reflect concurrent alterations in endothelial and prostatic markers. These associations should not be interpreted as evidence that NO inhibition causes PSA elevation, as NO production was not directly measured, and causality cannot be inferred. Furthermore, the observed reduction in citrulline and the resultant imbalance in the ratios of methylated arginine indicate a disruption in the arginine–NO flux; therefore, endothelial injury can serve as an early indicator of systemic Cd burden. These results are consistent with previous reports suggesting that Cd may substitute for Zn in enzymes, potentially influencing redox homeostasis, enzyme function, and receptor integrity. Such mechanisms have been described not only in vascular tissues but also in endocrine organs, including the prostate [18,22,23].

### 4.1. Toxic Metal Stress and Endocrine Dysfunction

Another key feature of this research project is the association of the PSA levels and methylated arginine metabolites (methylated arginine derivatives), suggesting crosstalk between endothelial dysfunction and prostatic activation. The presence of NO in the endothelium has an important function for the regulation of local blood flow and for promoting the growth of new capillaries (angiogenesis) and supplying nutrients (tissue perfusion) to the prostate. Mechanistically, elevations in ADMA and SDMA could plausibly reduce endothelial nitric oxide availability and contribute to microvascular or oxidative stress, processes that have been hypothesized to influence prostatic cellular activity. However, in the present study, this remains a theoretical possibility, as our data do not establish any causal link to PSA secretion [24,25,26]. Although PSA levels were significantly higher in the Cd-exposed group, the absolute values mostly remained within the clinically accepted reference range (<4 ng/mL). Therefore, these elevations likely reflect subclinical prostatic stress rather than pathological PSA increases. Nevertheless, even modest PSA elevations may indicate early epithelial or inflammatory responses requiring follow-up. As such, these results are consistent with prior research studies that have indicated that Cd exposure produces oxidative stress and inflammatory signaling in the prostate that may contribute to hyperplasia and carcinogenesis [27].

Cd has been reported to displace Zn in Zn finger motifs of transcription factors and metalloproteinases [28]. In the prostate, displacement of Zn from the androgen receptor (AR) and related DNA binding domains causes changes in the conformation of the receptor and its ability to regulate gene transcription, producing aberrant expression of genes that are involved in cell growth and differentiation [29,30]. The magnitude of ADMA and SDMA elevations in our cohort was comparable to levels reported in early chronic kidney disease, diabetes mellitus, and metabolic syndrome. These ranges are known to impair endothelial NO signaling, suggesting that Cd exposure induces endothelial dysfunction of clinically meaningful magnitude [31,32]. Additionally, Cd’s reaction with metallothioneins forms reactive Cd–thiolate compounds that will further enhance oxidative damage and endocrine disruption [33]. The interplay among oxidative imbalance, endothelial stress, and hormonal dysregulation may contribute to parallel alterations in PSA and methylated arginines, although the present data cannot determine directionality or causation.

### 4.2. Comparison of Exposure to Toxic Metals and Common Metabolic Features

Similarities exist in the toxicometabolic profile found here and those associated with exposure to arsenic, lead, and mercury, as they can each cause oxidative methylation stress and endothelial dysfunction [34,35]. However, Cd is unique because it can affect both the vascular endothelium and the endocrine system via Zn-dependent interference with proteins. Like arsenic, Cd exposure increases ADMA concentrations by inhibiting Dimethylarginine Dimethylaminohydrolase activity; however, it also affects Zn-dependent enzymes involved in hormone regulation. The similarities in the pathways affected by Cd and arsenic illustrate the broader mechanistic theme common among heavy metals: the interference with amino acid methylation cycles and redox-sensitive enzyme systems [36]. Interference with these systems not only suggests disruption of vascular homeostasis but also may lead to endocrine and reproductive dysfunction over time. Therefore, the combined metabolic alterations identified in this research—elevated ADMA and SDMA, decreased citrulline, and abnormal PSA levels—represent a common molecular profile for chronic metal toxicity; however, the distinct endocrine signature imparted by Cd is a distinguishing characteristic of this metal.

### 4.3. Systems-Level Thinking: Metabolic Flux and the Integration of the Omics

From a systems biology point of view, Cd exposure represents a metabolic disturbance with respect to flow through the arginine–NO pathway, an important node between nitrogen metabolism, oxidative balance, and cell signaling processes. The simultaneous increase in two competing substrates, ADMA and SDMA, together with decreased arginine: ADMA ratios, can be considered as a limiting metabolic flux bottleneck that limits NO production while increasing turnover of methylated arginine substrates. The combination of these variables into simple flux-balance analysis models could quantify loss in NO production and identify compensatory fluxes occurring in citrulline or urea cycle intermediates [37].

Ultimately, a multi-omics approach involving targeted metabolomics, redox proteomics, and transcriptomics would produce a toxicometabolic interaction map of Cd exposure that joins molecular perturbations arising from toxicity to clinically relevant biomarkers such as PSA [38]. This level of systems thinking will not only refine our current mechanistic understanding but also promote biomarker discovery for occupational health surveillance.

### 4.4. Toxicometabolic and Clinical Significance

These results merit future mechanistic research to support the findings of this study. Additional biomarkers that are specific to oxidative stress can be targeted to provide additional insight into Cd-induced oxidative injury, including 8-hydroxyguanosine as a measure of DNA damage and Malondialdehyde as a measure of lipid peroxidation, utilizing an Enzyme-Linked ImmunoSorbent Assay or Ultra Performance Liquid Chromatography–Tandem Mass Spectrometry. Global metabolomics and/or proteomics can identify new metabolic points of action affected by Cd exposure. Further, complementary in vitro studies using prostate epithelial cells or endothelial cells exposed to Cd will provide evidence for the biological mechanisms contributing to the changes observed in the biomarkers in this cohort.

Mechanistically, Cd may be associated with higher PSA levels and altered endothelial function, a shared upstream pathway involving oxidative stress-driven protein arginine N-methyltransferase activation, increased ADMA and SDMA production, and consequent nitric oxide depletion, potentially linking vascular injury and prostatic stress.

The present study highlights the clinical utility of assessing both vascular and endocrine biomarkers in populations exposed to Cd. The joint assessment of PSA, ADMA, and SDMA may be useful as a panel of early indicator tests of subclinical toxicity in exposed populations, which would allow preventive measures to be instigated before the onset of manifest disease [39,40,41]. Cd exposure is cumulative and irreversible, so metabolic profiling providing early warning gives an advantage in occupational risk management. From a public health point of view, the addition of methylated arginine profiling to occupational screening procedures would improve the early identification of workers at risk. This combined metabolic endocrine surveillance approach is in line with the objectives of precision medicine-type studies of metabolomics, where molecular fingerprints are correlated to individuals’ exposure histories and physiological outcomes [42].

## 5. Conclusions

Chronic occupational Cd exposure may point toward disruption of the nitric oxide axis, leading to elevated methylated arginine derivatives, in a pattern that is consistent with reduced bioavailable NO and subclinical endothelial dysfunction. Elevated levels of both PSA and dimethylarginine derivatives suggest that Cd exposure may be associated with alterations in both vascular and endocrine pathways, and may reflect potential effects on oxidative, metabolic, and/or hormonal processes.

The simultaneous measurement of ADMA, SDMA, and PSA can serve as an initial biomarker panel for detecting early systemic effects of Cd exposure in occupational environments. This approach also provides a foundation for metabolomics-based monitoring systems, enabling a systems-level understanding of Cd-induced metabolic disruptions. From a public health perspective, integrating this biomarker panel into occupational surveillance programs could facilitate earlier identification of at-risk workers, allow timely and targeted interventions, and ultimately reduce the long-term cardiovascular and urological burden associated with chronic Cd exposure. Moreover, the adoption of ADMA–SDMA–PSA monitoring may strengthen exposure-control measures and promote preventive strategies to protect workers and improve population-level health outcomes in sectors where Cd remains a persistent hazard.

The cross-sectional design of this study limits causal inference. Although strong correlations between urinary Cd, PSA, and methylated arginines were observed, temporal relationships cannot be established. Therefore, the findings should be interpreted as associations rather than direct mechanistic effects, and longitudinal studies are required to clarify causal pathways.

## Figures and Tables

**Table 1:** Comparison of demographic and biochemical parameter means between the Cd-exposed and control groups.

	Group	N	Mean	Std. Deviation	t	Sig. (2-Tailed)
Duration of exposure (years)	Exposed	75	8.72	2.84	0.057	0.954
	Control	75	8.69	2.79		
Age (years)	Exposed	75	40.43	6.22	−1.604	0.111
	Control	75	42.08	6.40		
Urine Cd- µg/L (µg/g creatinine)	Exposed	75	2.90 (5.48)	0.72 (1.22)	25.120	<0.001 *
	Control	75	0.50 (0.97)	0.40 (0.79)		
BMI (kg/m^2^)	Exposed	75	28.10	2.15	−1.074	0.284
	Control	75	28.52	2.60		
PSA (ng/mL)	Exposed	75	1.27	0.67	10.869	<0.001 *
	Control	75	0.37	0.25		
Arginine (µmol/L)	Exposed	75	87.12	41.91	−1.247	0.215
	Control	75	97.57	59.31		
Citrulline (µmol/L)	Exposed	75	15.37	7.99	−3.982	<0.001 *
	Control	75	19.59	4.52		
ADMA (µmol/L)	Exposed	75	0.29	0.12	4.283	<0.001 *
	Control	75	0.22	0.08		
SDMA (µmol/L)	Exposed	75	0.25	0.06	3.423	0.001 *
	Control	75	0.22	0.04		
Arginine/ADMA ratio	Exposed	75	339.40	182.05	−3.738	<0.001 *
	Control	75	483.27	279.19		
SDMA/ADMA ratio	Exposed	75	0.94	0.28	−3.389	0.001 *
	Control	75	1.09	0.28		

* Independent *t*-test was used.

## Data Availability

The raw data supporting the conclusions of this article will be made available by the authors on request.

## Supplementary Materials

The following supporting information can be downloaded at: https://www.mdpi.com/article/10.3390/toxics13121049/s1, Table S1: Relationship Among Occupational Cd Exposure, NO Pathway Metabolites, and Prostate Biomarkers.

## Abbreviations

The following abbreviations are used in this manuscript:

ADMA	Asymmetric Dimethylarginine
BMI	Body Mass Index
Cd	Cadmium
CVD	Cardiovascular Disease
DNA	Deoxyribonucleic Acid
ICP-MS	Inductively Coupled Plasma–Mass Spectrometry
LC-MS/MS	Liquid Chromatography–Tandem Mass Spectrometry
NO	Nitric Oxide
PCa	Prostate Cancer
PSA	Prostate-Specific Antigen
SD	Standard Deviation
SDMA	Symmetric Dimethylarginine
Zn	Zinc
