# Supplementary material for: Impact of Cadmium on Prostate-Specific Antigen and Endothelial Markers: A Risk for Prostatic Damage
Source: Toxics. 2025 Dec 4;13(12):1049. doi: 10.3390/toxics13121049 (PMC12737596; doi:10.3390/toxics13121049)
Supplement: Supplementary file 1 [file toxics-13-01049-s001.zip › toxics-3993501-supplementary.pdf]

Table S1. Relationship among occupational Cd exposure, NO pathway metabolites, and prostate biomarkers

n=150	Age	Urine Cd	BMI	PSA	Arginine	Citrulline (μmol/L)	ADMA (μmol/L)	SDMA (μmol/L)	Arginine/ADMA	SDMA/ADMA
Duration of exposure (years)	0.278**	0.239**	*	0.318**	*	*	*	*	*	*
Age	-	*	*	0.373**	*	*	*	*	*	*
Urine Cd(μg/L)		-	*	0.783**	*	-0.288**	0.338**	0.212**	-0.293**	-0.301**
BMI (kg/m2)			-	*	*	*	*	-*	*	*
PSA (ng/mL)				-	*	-0.283**	0.283**	*	*	-0.282**
Arginine (μmol/L)					-	*	*	*	0.808**	*
Citrulline (μmol/L)						-	*	*	*	*
ADMA (μmol/L)							-	0.541**	-0.409**	-0.746**
SDMA (μmol/L)								-	-0.288**	*
Arginine/ADMA									-	0.288**

*$p>0.05$ **Correlation is significant at the 0.01 level (two-tailed). Pearson correlation was used.
